# Low viscosity and high attenuation in MgSiO_3_ post-perovskite inferred from atomic-scale calculations

**DOI:** 10.1038/srep34771

**Published:** 2016-10-06

**Authors:** Alexandra M. Goryaeva, Philippe Carrez, Patrick Cordier

**Affiliations:** 1 Unité Matériaux et Transformations, UMR/CNRS 8207, Université de Lille 1, 59655 Villeneuve d’Ascq Cedex, France

## Abstract

This work represents a numerical study of the thermal activation for dislocation glide of the [100](010) slip system in MgSiO_3_ post-perovskite (Mg-ppv) at 120 GPa. We propose an approach based on a one-dimensional line tension model in conjunction with atomic-scale calculations. In this model, the key parameters, namely, the line tension and the Peierls barrier, are obtained from density functional theory calculations. We find a Peierls stress *σ*_*p*_ = 2.1 GPa and a line tension *Γ* = 9.2 eV/Å, which lead to a kink-pair enthalpy (under zero stress) of 2.69 eV. These values confirm that this slip system bears a very low lattice friction because it vanishes for temperatures above approximately 500 K under mantle conditions. In the Earth’s mantle, high-pressure Mg-ppv silicate is thus expected to become as ductile as ferropericlase. These results confirm the hypothesis of a weak layer in the D″ layer where Mg-ppv is present. Easy glide along [100](010) suggests strong preferred orientations with (010) planes aligned. Highly mobile [100] dislocations are also likely to respond to stresses related to seismic waves, leading to energy dissipation and strong attenuation.

The D″ region, which lies just above the core-mantle boundary (CMB), is of primary importance in geodynamics because it represents the thermal boundary layer, where heat is transferred by diffusion from the core to the convective mantle. Its viscosity plays a key role in controlling the amount of heat that can be extracted from the core, with significant implications regarding the dynamics of the mantle. The discovery of a phase transition from bridgmanite to a post-perovskite (ppv) phase at pressure (*P*) and temperature (*T*) conditions close to those of the CMB has opened new perspectives regarding the interpretation of the D″ structure and dynamics. This phase, which exhibits a very peculiar layered structure, has attracted much attention. Based on first-principles calculations, Ammann *et al*.^1^ showed that the diffusion of Mg^2+^ and Si^4+^ is extremely anisotropic in post-perovskite, with high diffusion rates along <100>. This finding has led to the idea that the D″ layer could be weaker than the overlying mantle due to high diffusion creep rates in ppv, although creep rates may be controlled by diffusion in the slowest direction^2^. However, this hypothesis is difficult to reconcile with the strong seismic anisotropy that is the signature of the D″ layer (as first observed in ScS^3^ and S_diff_^4^ phases) and is usually indicative of dislocation creep rather than diffusion creep.

Making the link between seismic anisotropy observations and putative crystal preferred orientations (CPO) in ppv is not straightforward. In horizontally propagating phases, the observation that horizontally polarized shear waves propagate faster than vertically polarized ones (V_SH_ > V_SV_) agrees with models involving dominant slip in (010) or (001) in ppv^5^,^6^. If only dominant slip on (100) seems to be excluded by seismological observations so far, the respective role of (010)^6^,^7^ and (001) slip^8^,^9^ remains debatable. From the mineral physics point of view, the hypothesis of slip in (010), which seems intuitive given the layered structure of post-perovskite, is difficult to assess experimentally. Indeed, observation of experimentally produced CPO in diamond cell experiments has also led to conflicting results^10^,^11^, which may result from textures formed during phase transformation^12^,^13^. Additionally, beyond the geometry of crystal plasticity (from CPO), there is no way currently to obtain experimentally quantitative data of the rheology of silicate post-perovskite under relevant *P*, *T* (and strain rate!) conditions of the D″.

Multiscale numerical modelling represents an alternative that is currently able to describe plasticity by dislocations of high-pressure minerals. Recent applications to wadsleyite^14^, ringwoodite^15^, periclase^16^ and bridgmanite^17^ have shown their ability to reproduce laboratory experiment data. The main implication of these studies (both numerical and experimental) is that pressure has a strong effect on the lattice friction opposed to dislocation glide. In bridgmanite, Hirel *et al*.^18^ showed that lattice friction increases monotonically throughout the lower mantle to reach, in the lowermost mantle, values of approximately 15 GPa. In this context, the behaviour of post-perovskite appears remarkable. Shearing the Si octahedral layers (for instance, with the [100](001) slip system) yields comparable values of lattice friction to those of bridgmanite^19^. However, shearing the structure parallel to the structural layering is much easier: lattice friction opposed to the glide of [100] screw dislocations in the (010) plane is one order of magnitude lower than that in (001). This is the reason why we focus on this slip system in the present study. Lattice friction describes the anisotropic mechanical resistance of the material at 0 K only; it is still necessary to model the thermal activation of dislocation glide to determine how lattice friction evolves in the conditions of the D″ layer.

In this work, we present a theoretical study based on full atomistic modelling of plastic deformation, demonstrating easy glide of [100](010) dislocations in MgSiO_3_ post-perovskite (Mg-ppv) at finite temperature and strain rate of the lowermost mantle.

## Results

### Dislocation core structure and Peierls barrier

**Figure 1:**
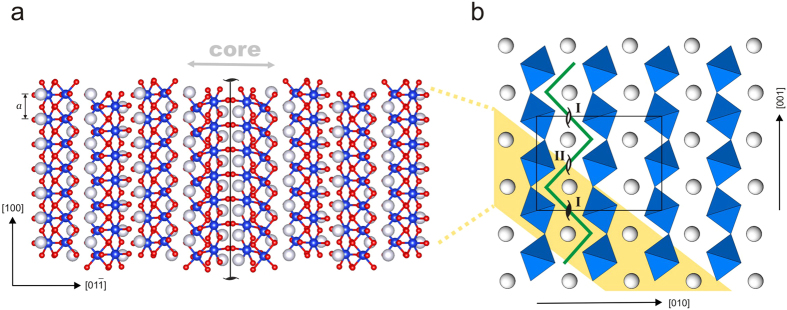
(**a**) Atomic structure of stable [100](011) dislocation core extracted from the two-cation thick (011) atomic layer highlighted in yellow on the right panel. (**b**) MgSiO_3_ post-perovskite structure viewed along [100]. Location of the low energy dislocation lines (I) and (II) is specified by the “screw” symbols. MEP of [100] dislocation gliding in the (010) plane (deduced from NEB simulations) is indicated by the green line. On the both panels, Si atoms and octahedra, forming rigid {010} layers, are shown in blue; Mg atoms, forming weaker {010} layers in grey. Red atoms on the left panel correspond to O atoms.

**Figure 2:**
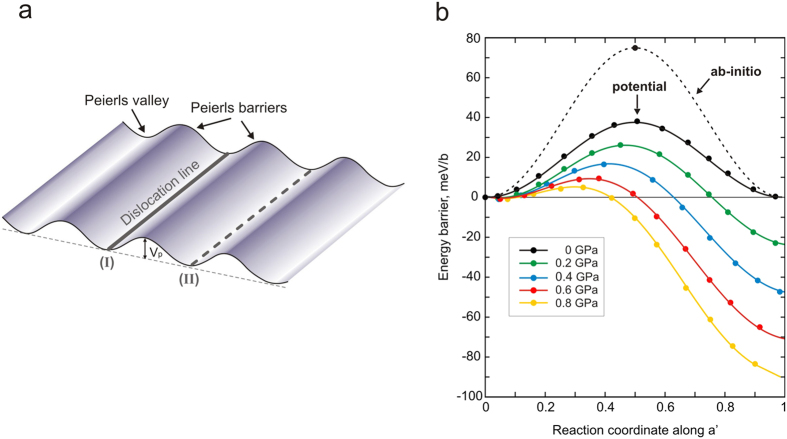
(**a**) Lattice friction: schematic illustration of a straight dislocation line gliding over the Peierls potential, which materializes the lattice friction. (**b**) Peierls barrier *V*_*p*_ calculated for [100](010), with DFT (dashed line) and pairwise potential (solid line). Evolution of the energy barrier with applied stress, i.e., *V*_*p*_ − *σ*_*a*_ *ba*′, deduced from NEB calculations with pairwise potential, is provided in colour (see the text for details).

The core structure of screw dislocations with the Burgers vector [100] is computed at the atomistic level using density functional theory (DFT). The atomic configuration of a straight dislocation line is shown in Fig. 1a. The stable core configuration is centred between two neighbouring Mg atoms and mainly spread in {011}. In (010), equivalent stable core configurations are found every ½[001], i.e. separated by a distance *a*′ of 3.07 Å. These cores correspond therefore to alternative variants labelled (I) spread in (011) and (II) spread in 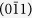, as displayed in Fig. 1b. Although DFT represents high accuracy calculations, we also employ a pairwise potential to compute the minimum energy path (MEP) between two stable cores associated with dislocation glide in (010) and to carefully investigate finite size effects (as described in the supplementary materials). The MEP between configurations (I) and (II) is computed using the nudged elastic band (NEB) algorithm^20^,^21^,^22^. The observed path reproduces the peculiar <011> zig-zag trajectory (Fig. 1b) reported in a previous atomic-scale study^19^. The maximum of the Peierls potential corresponds to the MEP dislocation image when the (011) glide trajectory switches to 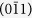. This high energy configuration is associated with 39 meV/*b* and 77.7 meV/*b* energy barriers for pairwise potential and *ab initio* simulations, respectively (Fig. 2b). Based on these results, the Peierls stress *σ*_*p*_ can be estimated from the maximum slope of the Peierls potential. Thus, DFT calculations lead to *σ*_*p*_ = 2.1 GPa, whereas the empirical potential simulations predict a lower value of 1 GPa (Fig. 2b). This apparent discrepancy is likely related to the known drawback of the pairwise potential parameterization used in this study. Indeed, both simulation techniques provide identical dislocation core structures, but the empirical potential underestimates the elastic stiffness coefficients *C*_*55*_ and *C*_*66*_ by a factor of 2 and, consequently, the corresponding anisotropic shear modulus *μ* (173 GPa vs 324 GPa) of the post-perovskite^23^. Qualitatively, one finds therefore the ratio *σ*_*p*_*/μ* ~5·10^−3^ regardless of the level of atomic description.

### Thermal activation and kink-pair mechanism

**Figure 3:**
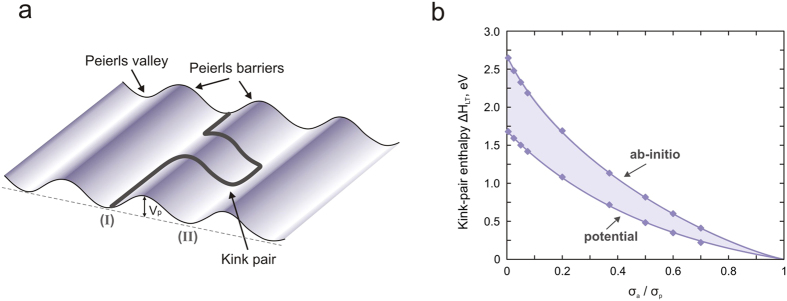
(**a**) Thermal activation of dislocation glide: schematic illustration of the kink-pair mechanism, which allows the dislocation line to pass from one stable position to the next at finite temperature. (**b**) Kink-pair formation activation enthalpy *H*_LT_ and its evolution with applied stress, computed with the data acquired from *ab initio* and semi-empirical simulations.

At finite temperature, the actual motion of a dislocation occurs through the nucleation and propagation of kink-pairs, i.e., a dislocation does not move as a straight line but partly bows out over the Peierls potential, as illustrated in Fig. 3a. Unlike common silicates, dislocation glide in (010) of Mg-ppv is associated with a relatively low energy barrier (in metals for example, a *σ*_*p*_*/μ* ratio of 10^−3^ is usually expected). Under an applied stress *σ*_a_, a straight dislocation will move upwards of the Peierls barrier to reach an equilibrium configuration from which a bulge can form. Energy minimization of the bowed configuration is obtained from a balance between the local forces on the dislocation, classically called the line tension, i.e., the force resulting from the Peierls barrier and the applied force. However, assuming that the fluctuation of the Peierls potential is small compared to the energy at rest leads to the standard line tension (LT) model for describing the kink-pair mechanism^24^,^25^,^26^. Within the LT formulation, a screw dislocation line can be represented as a 1D function *y*(*x*), which describes its position *y* in the glide plane at each *x* coordinate along the dislocation line (Fig. 3a). Then, the dislocation line enthalpy *H*_*LT*_ can be estimated according to the following expression:


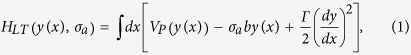


where *Γ* is the line tension (representing the stiffness of the dislocation line) and *V*_*p*_(*y*(*x*)) − *σ*_*a*_*by*(*x*) corresponds to the so-called “substrate enthalpy” in the 1D-Frenkel-Kontorova model^27^,^28^.

#### Computing line tension of a dislocation

To link the LT model with atomic-scale simulations, we follow the work of Dezerald *et al*.^29^ and discretize the integral in Eq. (1) into *n* segments {*Y*_*n*_} of length *b*:


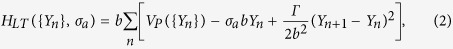


where the sum over *n* accounts for the periodic boundary condition along the dislocation line.

**Figure 4:**
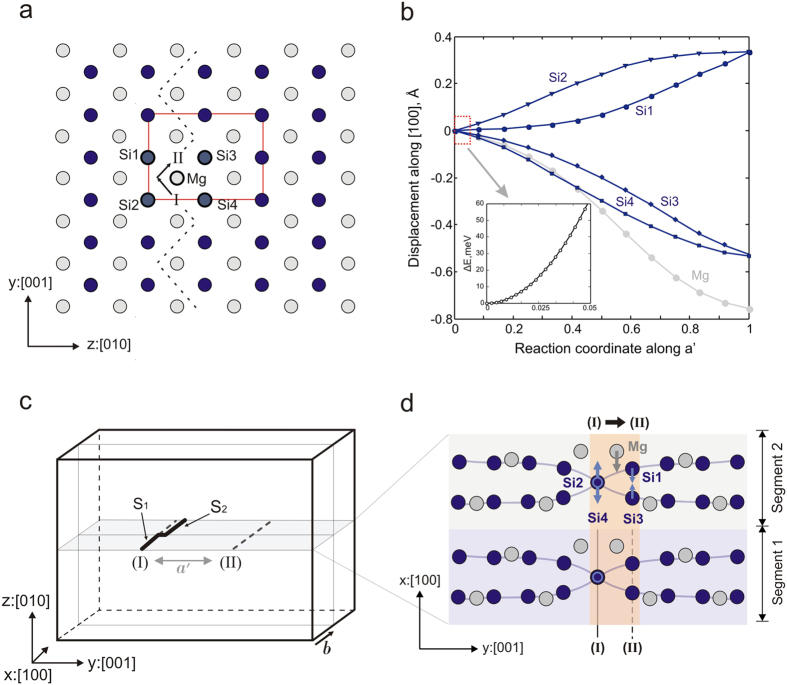
Line tension calculation scheme. (**a**) The five cations, four Si and one Mg, that exhibit the biggest displacements along [100] during dislocation glide are labelled here. Si atoms are indicated by blue spheres, Mg by grey, the anion sublattice is left out for clarity, the dashed line indicates the MEP followed by the dislocation during its glide, and the unit cell is indicated by the red rectangle. (**b**) Atomic displacements along [100], deduced for the five selected cations from NEB simulations. The plot provides displacements for a screw dislocation with positive Burgers vector; in the case of negative Burgers vector, atomic disposition occurs in the inverse way. (**c**) Schematic illustration of dislocation line bending, mimicking the instant of kink-pair formation, in a 2*b* simulation cell. Glide plane (010) is highlighted in grey. (**d**) Local structure of the cation sublattice produced by the screw dislocation (I) in the (010) plane. To create a bow-out, degrees of freedom along [100] are restricted for the cations located in the pink area: in segment *S*_*1*_, the selected atoms are fixed to their regular positions in the Peierls valley (I), while the corresponding atoms of segment *S*_*2*_ are forced to follow up to 5% of the MEP along *a*′ towards the next Peierls valley (II), as indicated by the arrows. The associated increase in elastic energy ∆*E* is provided in (**b**).

To compute the line tension *Γ*, the energy cost associated with a dislocation bow-out consistent with the first stage of kink-pair formation should be estimated. Bending a dislocation line requires breaking up the 1*b* translational symmetry of the simulation cell. Thus, the length of the supercell along the dislocation line is increased up to 2*b*. We consider a dislocation line that consists of two segments of length *b*: segment *S*_*1*_ remains in the Peierls valley (I), while the other segment *S*_*2*_ bows out towards the next valley (II), as shown in Fig. 4c. To compute this process at the atomic scale, the evolution of atomic displacements *Δx* along [100] during the dislocation glide from (I) to (II) is analysed, relying on MEP structural information obtained from NEB simulations. Dealing with a complex material, we mostly focus on the cation sublattice and allow anions to adapt to the local displacement of cations. Along the MEP, one Mg and four Si atoms exhibit the largest displacements *Δx* along the dislocation line among the cations in the crystal (Fig. 4a,b). The selected Mg atom is located directly between the two Peierls valleys and bears the maximum displacement amplitude (approximately 0.8 Å). The *Δx* amplitudes of the four Si atoms are two times smaller (Fig. 4b). Once the evolution of atomic positions along the MEP is defined, one can create the dislocation bow-out while applying the exact displacements *Δx* consistent with the MEP (at zero stress) to the selected atoms belonging to the segment *S*_*2*_ of a dislocation line (Fig. 4d). The corresponding cations of segment *S*_*1*_ are fixed to their regular positions in the Peierls valley, constraining the degrees of freedom along the dislocation line. The computed change in energy, 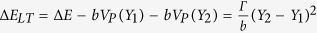, related to the gradual disposition of line segment *S*_*2*_ is shown in Fig. 4b as a function of the reaction coordinate along [001]. Fitting the curvature of quadratic function *ΔE*_*LT*_ provides the line tension *Γ* = 9.2 eV/Å for DFT simulations (vs *Γ* = 7.1 eV/Å for empirical potential). The anisotropic line tension *Γ*_*el*_ = 2.08 eV/Å, calculated within Stroh formalism^30^ using the set of elastic constant *C*_*ij*_ of Mg-ppv^31^ computed with the same generalized gradient approximation (GGA) and pseudopotentials, is notably lower. Previous studies of bcc metals based on a similar simulation approach for computing LT at the atomic scale^29^,^32^ report comparable discrepancies between the LT values predicted atomistically and from elastic theory. Indeed, the latter does not account for the large effect of the dislocation core contribution, which leads to drastically underestimated *Γ*_*el*_ values.

#### Kink-pair activation enthalpy

Once line tension *Γ* is computed at atomic scale and the Peierls barrier *V*_*P*_ is known, the equilibrium kink-pair shape at a given stress and the corresponding critical kink-pair enthalpy *H*_*LT*_ can be calculated using Eq. (1). To solve Eq. (1), we rely on a trial function *y*(*x*) that describes the equilibrium shape of a symmetric kink-pair based on a combination of hyperbolic tangents^28^:


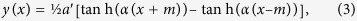


where, as previously mentioned, *a*′ is the periodicity of the Peierls potential and *α* and *m* are variable parameters.

The saddle point on the *H*_*LT*_(*α*,*m*) energy landscape ultimately defines both the enthalpy *H*_*LT*_ and the equilibrium kink-pair configuration *y*(*x*). As illustrated in the Supplementary materials, kink-pair configurations are characterized by extremely large widths in the range of 35–40*b* (approximately 100 Å) resulting from the very low Peierls barrier. With applied stress, this width changes moderately while the height of the kink-pair decreases rapidly as the straight part of the dislocation line moves upwards along the Peierls barrier. Note that such a wide kink shape justifies our choice of the LT model with respect to unreasonable direct atomistic computation of bowed configuration lines (which would require too many atoms). Figure 3b shows the computed enthalpy *H*_*LT*_ as a function of applied stress *σ*_*a*_. As one expects from dislocation theory^33^, the kink-pair enthalpy is maximum under zero stress, with a value of 2.69 eV, corresponding to twice the energy of a single kink *H*_*k*_, and it vanishes when the applied stress is equal to the Peierls stress. The normalized kink-pair enthalpy *2H*_*k*_*/μb*^3^ is found to be approximately 5 · 10^−2^, confirming the relatively low lattice friction borne by the [100](010) slip system in post-perovskite.

## Discussion

The dislocation core structure computed here confirms the spreading of the [100] screw core in {011}, as observed in previous semi-empirical simulations^19^. The extension of the core (with a half-width of approximately 1.8 Å) is found to be in reasonable agreement with the first results of the dislocation core determined using the Peierls-Nabarro model^31^. In Carrez *et al*.^31^, the classical PN model, based on first-principles calculations (with the same GGA approximation and pseudopotentials as in this study) of generalized stacking fault energy, showed that the [100] screw dislocation should be compact in {011}, with a half-width of 1 Å. Discrepancies in core size between the present results and those of the PN model can be largely attributed to the GSF calculations method. As shown in Goryaeva *et al*.^19^, the GSF computed by Carrez *et al*.^31^ involved atomic layers above the actual spreading layer of the core, delimited by neighbouring Mg rows. Consequently, the GSF energies used in the PN model were overestimated, leading to a narrower core. Nevertheless, note that despite a spreading in {011}, the easiest glide plane of [100] screw dislocations is (010). This is the result of a glide alternating between (011) and 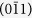, defining a global macroscopic glide plane (010). This non-standard behaviour could be evidenced only by full atomistic calculations. Based on calculation of the maximum height of the Peierls potential, we find a Peierls stress of 2.1 GPa, which is almost forty times smaller than the value reported in Carrez *et al*.^31^. The reason for this discrepancy is that the PN calculation of *σ*_*p*_ did not rely on the right lattice periodicity *a*′. The lattice periodicity *a*′ is found here to be ½[001] without any ambiguities based on the computation of the exact core energy. Moreover, even considering the correct lattice periodicity for the Peierls potential, the PN model would certainly not be accurate in evaluating the Peierls potential for such a rearrangement of atoms around the line during the glide. Indeed, the dislocation glide process (through a zig-zag scheme) violates one of the intrinsic hypotheses of the PN model, which is that the Peierls potential is evaluated in the plane of dislocation core spreading.

In post-perovskite, the Peierls stress of [100](010) glide at 120 GPa is remarkably low. This result is consistent with the observation of [100](010) dislocations in several experimental studies of low-pressure CaIrO_3_ and CaPtO_3_ post-perovskite analogues^34^,^35^,^36^,^37^. Because Mg-ppv is unquenchable, experimental studies remain scarce^10^,^11^. From the experimental point of view, in CaIrO_3_ post-perovskite, the few TEM studies^36^ do not show strong evidence of lattice friction for [100] dislocations (as observed for olivine deformed at low temperature, where dislocation lines tend to be aligned along a particular direction). This can be supported by several results found here, i.e., the low normalized values of Peierls stress and kink-pairs enthalpy, and from the computed line tension. As recently demonstrated for perovskite material^17^, kink-pair enthalpy evolution as a function of stress can be used to infer the evolution of the critical shear stress for dislocation glide as a function of temperature:


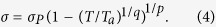


In Equation (4), *p* and *q* describe the evolution of the kink-pair enthalpy through the following relationship: Δ*H*_*LT*_(*σ*_a_) = 2*H*_k_ (1 − (*σ*_a_/*σ*_p_)^*p*^)^*q*^. Temperature *T*_*a*_, often called “athermal temperature”, corresponds to the critical temperature at which lattice friction vanishes. Generally, kink-pair energy 2*H*_*k*_ scales with *T*_*a*_ according to *2H*_*k*_ = *CkT*_*a*_, where *k* is the Boltzmann constant; *C* is a function of a strain rate *ε*′, of the dislocation density *ρ*, and of the kink geometry (for more details see the corresponding section of the Supplementary Materials). *C* is classically found in a range of 20–30 (this is verified in metals^38^ and also in oxides^39^). Therefore, taking 2*H*_k_ = 2.69 eV, *p* = 0.73 and *q* = 1.31 from the DFT calculations (considering experimental conditions, i.e., strain rates of 10^−5^ s^−1^ and dislocation density of 10^12^ m^−2^), we find that lattice friction vanishes if the temperature is raised above 1,100 K.

**Figure 5:**
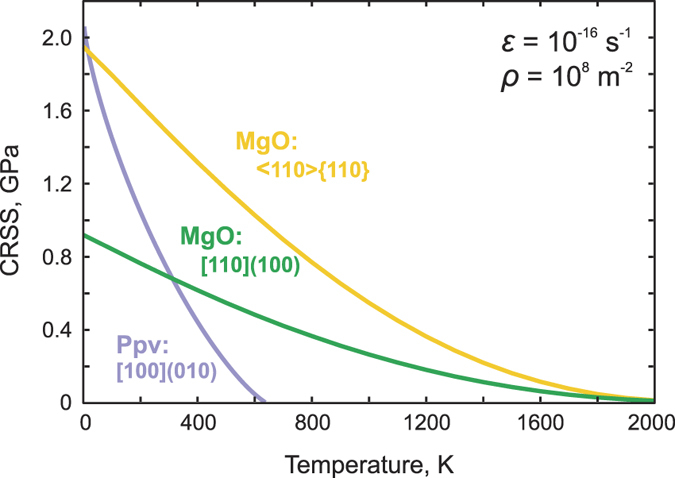
Evolution of the critical resolved shear stress (CRSS) with temperature for [100](010) in Mg-ppv at 120 GPa, compared with that for ½[110](100) and ½<110>{110} in periclase16 at 100 GPa. CRSS for post-perovskite is computed based on the data inferred from DFT simulations. Strain rate 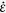 and dislocation density *ρ* correspond to the lower mantle conditions (see the text for details).

More importantly, as demonstrated recently for bridgmanite^17^, the previous equation can be used in Earth mantle conditions by adjusting the scaling factor of *T*_*a*_ to strain rates characteristic for convection in the Earth’s mantle. Assuming a typical value of 10^−16 ^s^−1^, the corresponding temperature evolution for the critical stress for the glide of [100] dislocations is as shown in Fig. 5.

### Implications

Our results on dislocation glide in Mg-ppv, including certain unexpected results, shed new light on the rheology of high-pressure mantle phases. Indeed, all recent studies, either experimental or theoretical, of wadsleyite^14^,^40^,^41^,^42^, ringwoodite^15^,^43^,^44^, periclase^16^,^45^ and bridgmanite^17^,^46^ consistently show that pressure in the transition zone and lower mantle range leads to a significant increase of lattice friction, which inhibits dislocation glide as a strain-producing mechanism. In particular, these results have important implications regarding the (non-)formation of seismic anisotropy from the deformation of the above-mentioned phases. In this context, it is surprising to find that crystal chemistry and the formation of a layered structure can lead to a completely different behaviour. Our results demonstrate that the presence of weak {010} Mg-layers containing a very short <100> lattice repeat of 2.5 Å leads to dislocation structures that can easily glide. We find that lattice friction is overcome at a critical temperature *T*_*a*_ far below the temperatures expected in the D″ layer (3,700–4,400 K^47^,^48^). This finding has several unexpected consequences. The relative ease of slip between Mg-ppv and periclase suggests that the latter could become the stronger phase in the D″ layer. We are not yet in a position to fully establish this fact because additional deformation mechanisms must be activated in Mg-ppv to ensure compatibility of plastic deformation in an aggregate. However, the fact that diffusion is also fast in this phase^1^ suggests that complementary deformation mechanisms involving diffusion should be easily activated. Mg-ppv being the dominant phase in this assemblage, it is expected that the D″ layer in regions dominated by the Mg-ppv should exhibit a very low viscosity compared to the overlying mantle.

The implications of such a low viscosity layer have already been considered and discussed^49^,^50^,^51^. The way that slabs behave when ultimately reaching the CMB is clearly affected, as is the broad dynamics of the CMB. However, the strongest implication is probably the enhancement of heat transfer from the core across the CMB, as earlier predicted by Buffett^49^ and more recently investigated numerically^50^,^51^. The most testable implication of our results is, of course, the strong (010) crystal preferred orientation, which should develop upon flow in this weak layer. This is an important parameter because the D″ layer has long been recognized as being highly anisotropic. Although no consensus has yet been reached (see, for instance, Cottaar *et al*.^9^), our finding that Mg-ppv exhibits dominant easy glide along (010) is consistent with the most recent studies of Nowacki *et al*.^6^ and Ford and Long^7^.

In addition to a low viscosity, a low lattice friction in Mg-ppv may have important implications regarding seismic wave attenuation. A seismic (body) wave corresponds to strains in the range of 10^−8^–10^−6^, with periods in the range of 1–10 s. These values correspond to stresses of a fraction of a MPa at most, applied at a strain rate of 10^−6^ s^−1^ or lower. Under these conditions, the athermal temperature *T*_a_ will be greater than in Fig. 5, but, in any case, lower than 1,400 K (the value corresponding to a strain-rate of 10^−5^ s^−1^, constraining the dislocation density at 10^8^ m^−2^; see supplementary Figure S5). This result shows that Mg-ppv will be in the athermal regime under seismic loading conditions at temperatures of the D″ layer, with dislocations moving freely without lattice friction.

This situation has not been considered up to now for seismic attenuation because most discussions have been driven by the example of olivine^52^,^53^. Olivine exhibits lattice friction; thus, dislocations are prescribed to stay in their Peierls valleys, and dislocation damping can only result in a limited contribution from kink migration^52^,^53^. For this reason, the most important source of attenuation in olivine has been linked to diffusionally assisted grain boundary sliding^54^.

Under an applied stress *σ*_a_ and without lattice friction, a dislocation segment of length *l* will bow out with a curvature 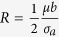. This bowing gives rise to a reduction of the effective shear modulus, called the *modulus defect* or *relaxation strength*: 
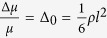
, where *ρ* is the dislocation density^55^. Assuming that *l* scales with 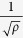, one can conclude that Δ_0_ could be as high as 15% (as an upper bound). Consequently, a shear wave travelling through the post-perovskite containing dislocations could encounter a maximum velocity reduction 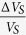 approximately 7% compared to the ideal structure. In this regime, dislocation damping can be described using the vibrating string model^55^ which assumes that under an applied alternating stress, a dislocation characterized by a line tension can execute forced vibrations like a vibrating string. An alternating stress, such as the one associated with a seismic wave, will result in damping and energy dissipation. This model has two important consequences. At sufficiently high frequency, there exists a peak in tan*ϕ* versus *ω* with a resonance at *ω*_0_ such that 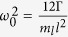. The frequency *ω*_0_ is a function of *Γ*, i.e., the line tension of the dislocation, as previously computed, and of *m*_*l*_, the effective mass per unit length of the dislocation line. This effective mass *m*_*l*_ can be computed by summing the squared displacements *dq*_*i*_ of all atoms *i* in a simulation cell in which a dislocation has moved from one Peierls valley to the next one, i.e., by *dQ* = *a*′, using the following expression^56^:


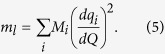


In the previous expression, *M*_*i*_ corresponds to the mass of atom *i*; the effective mass of the dislocation thus incorporates the kinetic energy of surrounding atoms as if they were to respond adiabatically to dislocation motion. Computed from different cell sizes to account for finite size effects, we find (in units of atomic mass per unit length) *m*_*l*_ ~ 9.7 *u*/Å, which results in *ω*_0_ ~ 3.3·10^4^* s*^*−1*^. This frequency is higher than that of seismic waves, but it could allow experimental verification in the laboratory. At lower frequencies corresponding to seismic waves, the internal friction is proportional to *ρl*^4^ and to the frequency *ω*.

Our proposition of post-perovskite being highly attenuating is consistent with the report of higher attenuation in the D″ by Anderson and Hart^57^ and Lawrence and Wysession^58^. However, attenuation in the D″ layer is still not well constrained; this issue deserves more attention in the future to verify our prediction.

## Conclusions

In this study, we modelled the thermally activated mobility of [100](010) slip in Mg-ppv using the line tension model in conjunction with atomic-scale simulations.

We show that under pressure, temperature and strain-rate conditions of the lowermost lower mantle, there is no lattice friction opposed to the glide of these dislocations. This easy glide of [100](010) dislocations has several implications:

- Although the exact viscosity of Mg-ppv cannot be calculated from a single slip system, we can predict a weak behaviour comparable with, if not weaker than, that of periclase.

- This conclusion supports the scenarios that involve a weak layer in the D″ layer with, in particular, enhancement of heat transfer from the core.

- Easy glide along (010) suggests development of marked crystal preferred orientations characterized by alignment of the (010) planes.

- The high mobility of dislocations allows for energy dissipation (vibrating string model) when a seismic wave travels through dislocation-bearing Mg-ppv. We predict that deforming Mg-ppv should be characterized by strong seismic attenuation.

## Methods

In this work, we employ an effective combination of the first-principles simulations with pairwise potential modelling. All simulations are performed with an external pressure of 120 GPa. Unit cell parameters for a Mg-ppv perfect crystal (*Cmcm*, Z = 4) at relevant conditions are *a*_*1*_ = 2.474 Å, *a*_*2*_ = 8.112 Å, *a*_*3*_ = 6.139 Å for the first-principles simulations and *a*_*1*_ = 2.521 Å, *a*_*2*_ = 8.124 Å, *a*_*3*_ = 6.050 Å for the semi-empirical calculations.

*Ab initio* calculations are performed based on DFT within the GGA, as derived by Perdew and Wang^59^, and the all-electron projector augment-wave (PAW) method, as implemented in VASP code^60^,^61^. The outmost core radius for Mg, Si and O is 2.0, 1.9 and 1.52 au, respectively. To achieve computational convergence, we apply a plane-wave cut-off of 600 eV. The first Brillouin zone is sampled using the Monkhorst-Pack scheme^62^, with a 10 × 1 × 1 k-point grid for 1*b* simulation cells containing 360 atoms (the exact geometry is described below) and with a 4 × 1 × 1 grid for 2*b* cells containing 720 atoms. The convergence energy is 10^−3^ meV/atom.

Atomistic simulations within the semi-empirical approach are carried out using the Buckingham form of a pairwise potential, with the parameterization derived by Oganov *et al*.^63^ for MgSiO_3_ perovskite. Transferability of this parameterization has been previously validated for modelling ground state properties and defects in the post-perovskite phase^23^. Molecular statics simulations are performed using the program package LAMMPS^64^, which relies on Ewald summation methods for Coulombic interactions. Optimization of dislocation core configurations are performed using a conjugate-gradient (cg) algorithm, followed by a Hessian-free truncated Newton (hftn) algorithm, until the maximum force on an atom drops below 10^−9^ eV/Å (1.602·10^−18^ N). The NEB simulations are performed via fire damped dynamics, as required by the minimization procedure implemented in LAMMPS. The MEP is sampled with 24 points (configuration images), which are bounded with a spring constant of 0.1 eV/Å.

All simulations are performed by employing a quadrupole arrangement of screw dislocations in fully periodic atomic arrays. Such simulation cells contain two dislocations with positive and two dislocations with negative Burgers vectors arranged as a rectangular checkerboard pattern. This geometry allows for cancelling the long-range displacement field produced by a dislocation^65^ and ensures that interaction of the dislocations remains at a quadrupolar level and that the net force on each core is zero due to the periodic arrangement^66^. The supercell is designed in such a way that [100] dislocation lines are parallel to *x* and [010] and [001] crystallographic directions are aligned with *z* and *y*, respectively. For core energy calculations and evaluation of the Peierls potential, the designed atomic systems are as thin as a single Burgers vector *b* along *x*, i.e., dislocation lines are straight and infinite due to the periodic boundary conditions. Because computation of line tension *Γ* requires bowing out the dislocation line and breaking the translational symmetry along *x*, we increase the length of the supercell along the dislocation line and employ 2*b* geometry, following the strategy proposed by Rodney and Proville^67^. For DFT simulations, we employ the smallest possible atomic array of 36 Å × 48 Å, with quadrupolar arrangement of dislocations, which is further reduced by half to a non-rectangular (but still fully periodic) cell containing a dislocation dipole, following the procedure described by Bigger *et al*.^66^. By applying periodic boundary conditions to such a dipole, the rectangular checkerboard pattern arrangement of dislocations (identical to that in the original rectangular cell containing four dislocations) is explicitly reproduced. The reduced simulation cells employed in this work for DFT simulations contain 360 and 720 atoms for 1*b* and 2*b* geometry, respectively. For the simulations performed with the pairwise potential, the size of the atomic arrays, containing a quadrupole of <100> screw dislocations, is gradually increased along *y* and *z* to track the size effect on the computed substrate enthalpy *V*_P_ and line tension *Γ*. A typical rectangular simulation cell has a size of 97 Å × 97 Å and 3840 and 7680 atoms in the case of 1*b* and 2*b* geometry, respectively.

Finally, for periodic arrangements of opposite Burgers vector dislocations, we compute the elastic interaction term, in accord with anisotropic elastic theory^68^, and subtract from the energy computed via the NEB method. The exact location *Y*_c_ of each dislocation image is defined through analysing the relative atomic displacements near the dislocation cores, which are further used to compute the disregistry function *S*(*Y*) using the following expression^69^:


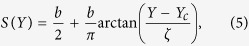


where *b* is the Burgers vector, *Y*_c_ is the coordinate of a dislocation centre, and *ζ* is an adjustable parameter.

## Additional Information

**How to cite this article**: Goryaeva, A. M. *et al*. Low viscosity and high attenuation in MgSiO_3_ post-perovskite inferred from atomic-scale calculations. *Sci. Rep.* **6**, 34771; doi: 10.1038/srep34771 (2016).

## Supplementary Material

Supplementary Information
