# Supplementary material for: Low viscosity and high attenuation in MgSiO3 post-perovskite inferred from atomic-scale calculations
Source: Sci Rep. 2016 Oct 6;6:34771. doi: 10.1038/srep34771 (PMC5052529; doi:10.1038/srep34771)
Supplement: Supplementary Information [file srep34771-s1.pdf]

SUPPLEMENTARY MATERIALS

Low viscosity and high attenuation in $MgSiO_3$ post-perovskite inferred from atomic-scale calculations

Alexandra M. Goryaeva, Philippe Carrez, and Patrick Cordier[*]

Unité Matériaux et Transformations, UMR Université de Lille1-
CNRS 8207, 59655 Villeneuve d'Ascq cedex, France

[*]Corresponding author: patrick.cordier@univ-lille1.fr

S1. Dislocation core structures

In this work we employ DFT simulations (see models and methods) to compute stable [100] dislocation cores (corresponding to the dislocation lines lying in the Peierls valley) and high-energy [100] core configurations (corresponding to the maximum of the Peierls potential V_P(max)). Location of the latest in the crystal structure was chosen based on the dislocation geometry predicted by NEB calculations preformed with the pairwise potential, described below.

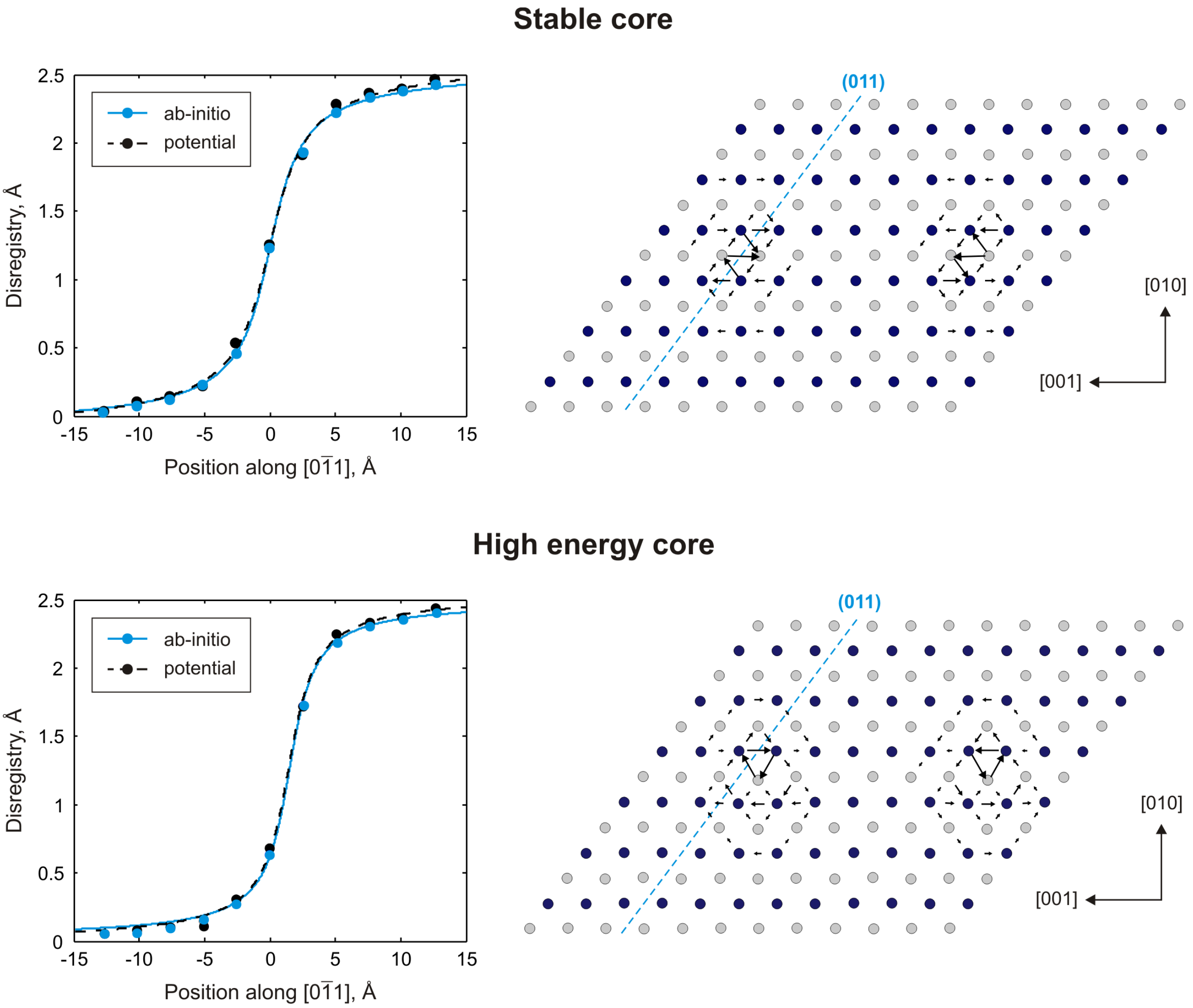

Figure S1. Right: DD-plot maps of [100] dislocation cores computed with DFT. Si atoms are shown in blue; Mg atoms – in gray; anion sublattice is left out (for clarity). The arrows between atoms correspond to the [100] component of the relative displacements of the neighboring atoms produced by the dislocation. The length of the arrows is proportional to the magnitude of these components.
Left: Disregistry functions (eq. 5) computed for cation sublattice in (011) plane (shown with dotted blue line on the DD-plot maps) in comparison with the corresponding functions computed for the [100] dislocation cores from the pairwise potential simulations (performed for atomic arrays of similar size)

S2. NEB calculations with pairwise potential

In this work, nudged elastic band (NEB) simulations are performed via fire damped dynamics, as required by the minimization procedure implemented in LAMMPS (Henkelman & Jonsson 2000; Henkelman et al. 2000; Nakano 2008). The minimum energy path (MEP) is sampled with 24 points (configuration images) which are bounded with a spring constant of 0.1 eV/Å. Simulations are performed using 1b simulation cell with quadrupolar arrangement of dislocations. In the initial configuration, all 4 dislocations are equidistant and located at Peierls valleys (I); in the final state two dislocations of the opposite sign remain in the initial Peierls valleys (I) while two other dislocations are displaced to the next valleys (II). In order to account for the effect of applied stress σ_a and increase the corresponding σ_{xz} stress component in the yz glide plane, a strain component ε_{xz} resulting in a certain σ_a value is then applied. For each atomic configuration along the MEP, the elastic interaction term is estimated in accord with anisotropic elastic theory (Clouet 2011) and subtracted from the energy computed with the NEB method.

Critical stress and minimum energy path

Critical stress σ_c required to overcome the energy barrier ΔH_P, which includes contributions of the Peierls potential V_P and the work of the applied stress σ_a (see Fig.2a), can be calculated as maximum of dH_P/b^2dy. With no stress applied, σ_c ~1 GPa corresponds to the Peierls stress. The estimated σ_c are shown as a function of applied stress σ_a on Fig. S2.1. Linear relation between σ_c and σ_a indicates that the Peierls stress σ_p does not strongly depend on applied stress.

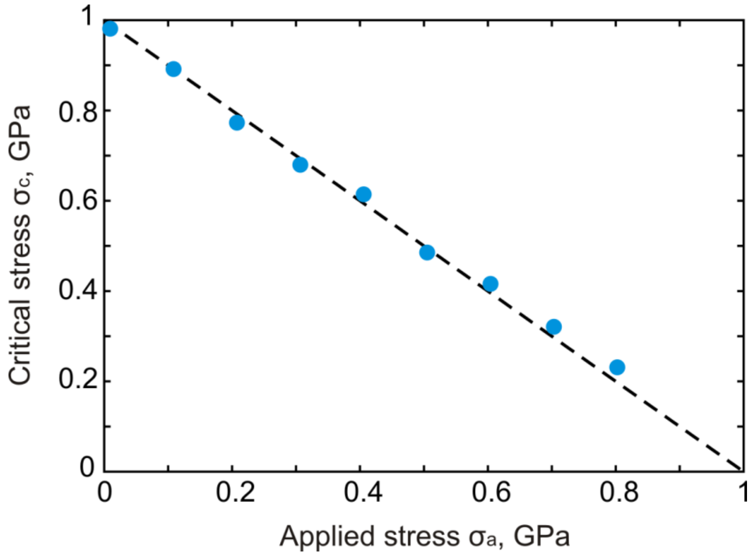

Figure S2.1 Evolution of the critical stress σ_c with applied stress σ_a. Data is derived from the simulations performed for the 97 Å × 97 Å × 1b cell containing 3840 atoms

Figure S2.2 illustrates the observed minimum energy path (MEP) of a straight [100] dislocation gliding. With no stress applied, this path reproduces the peculiar <011> zig-zag trajectory reported in previous atomistic study (Goryaeva et al. Part2 2015). The maximum peak of the Peierls potential is related to the MEP dislocation image when (011) trajectory switches to (0-11). Due to the very low lattice friction (~1 GPa), once $\sigma_a \geq 400$ MPa is applied, dislocation starts climbing the potential and equilibrium position of the dislocation line is displaced from the valley (I) towards (II) by Δz along [010] and by Δy along [001] (Fig. S2.2). This means, at applied stress, the path which [100] dislocation follows in (011) is not any more symmetric to that in (0-11). However, as it is shown above, this asymmetry does not have a strong effect on the critical stress needed to overcome the energy barrier.

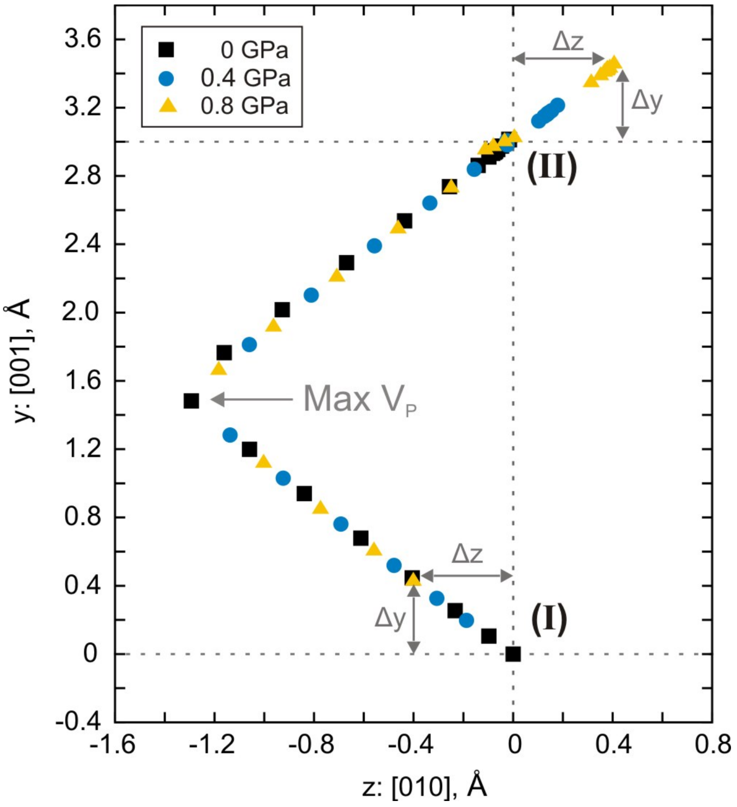

Figure S2.2. Minimum energy path of a straight [100](011) screw dislocation gliding from the Peierls valley (I) to (II) and its evolution with applied stress σ_a. Trajectory is defined from the NEB simulations performed for the 97 Å × 97 Å × 1b cell containing 3840 atoms

Size effect

For the NEB simulations, size of simulation cells was gradually increased until there is no effect on computed Peierls barrier (see Table S2). The optimal size of a supercell is found to be 97

Å × 97 Å × 1b. However, for DFT simulations, we are restricted to the smallest atomic array (marked with a star in Table S2). Relying on the observed tendency for the pairwise potential, one can expect that the energy barrier computed with DFT will be ~10% underestimated.

Table S2

Simulation cell size	Number of atoms	Maximum V_P
36 Å × 49 Å × 1b*	720	34.8 meV
81 Å × 85 Å × 1b	2800	37.5 meV
97 Å × 97 Å × 1b	3840	38.0 meV
146 Å × 145 Å × 1b	8640	38.1 meV

* Also employed for DFT simulations

S3. DFT simulations of the Peierls barrier V_P and line tension Γ

The smallest quadrupolar cell containing 720 atoms (Table S2) is reduced into a twice smaller dipole (Fig. S1) as suggested by (Bigger et al. 1992). However, even for the reduced cell with 360 atoms, performing direct NEB simulations with DFT appears to be extremely computationally expensive. Alternatively, in order to estimate the Peierls barrier from the first principles, we reconstruct the high-energy dislocation core associated with the maximum of the Peierls barrier (provided by NEB simulations with empirical potential) and employ it for DFT simulations. In this work, we rely on the values computed with 10 × 1 × 1 k-point mesh. Assuming the trend of the size effect to be similar to that observer for the pairwise potential (+10%), the corresponding DFT energy barrier can be expected to be close to 84.5 meV/b which results in the Peierls stress of ~2.3 GPa.

Since DFT and pairwise potential modelling provide very close configurations of dislocation cores (Fig. S1), we employ the MEP provided by NEB simulations (Fig.4b) to mimic a dislocation line bending both with DFT and pairwise potential. After structural relaxation, the exact location of segments S_1 and S_2 are defined through computing the disregistry function (eq. 5) at each step of the line “bending”. Figure S3 illustrates the energy cost computed for the 2*b* simulation cells containing 720 atoms with DFT and pairwise potential. Based on the pairwise potential, the size effect for bigger atomic arrays is found to be about +2%.

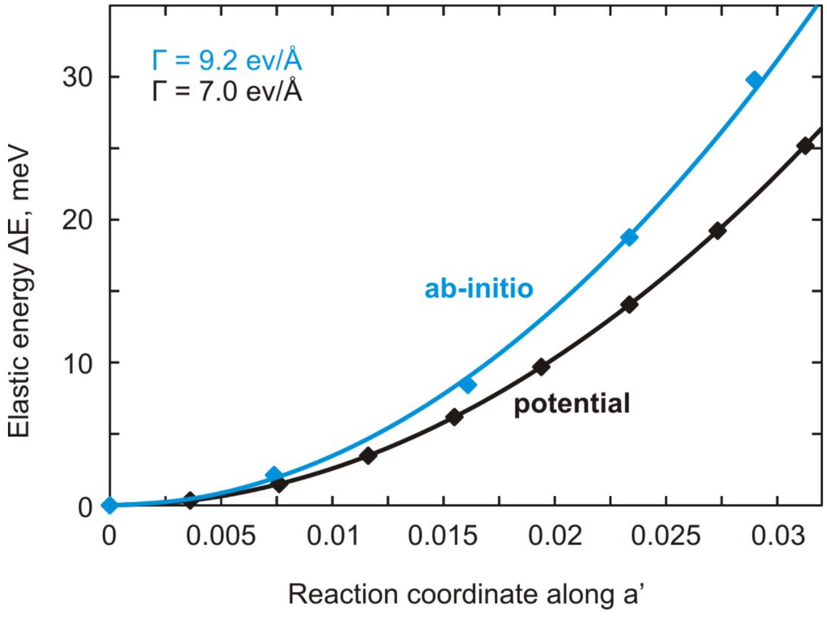

Figure S3. Elastic energy Δ*E* inferred from pairwise potential and DFT simulations performed for the cell containing 720 atoms. The corresponding line tension *Γ* values are provided on the plot

S4. Kink Shape

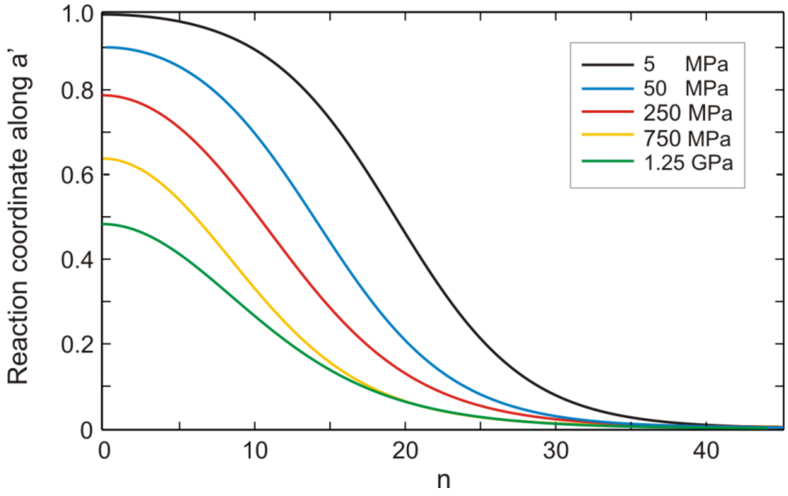

Figure S4. Kink shape computed based on the data acquired from DFT simulations. Along horizontal axis, n corresponds to number of Burgers vectors *b* = 2.474 Å

S5. Evolution of CRSS with Temperature

Combining Orowan's equation with the assumption of a dislocation velocity controlled by kink-pair nucleation, the shear strain rate $\acute{\varepsilon}$ can be defined as:

$$\acute{\varepsilon} = \rho b\bar{v} = \frac{\rho L a' b^2 \nu_D}{2w^2} exp\left(\frac{-\Delta H_{2k}(\sigma)}{kT}\right), \qquad (S1)$$

where ρ is the dislocation density; $\bar{v}$ is the dislocation glide velocity; b is the full Burgers vector length; a' stands for periodicity of the Peierls potential; w corresponds the kink-pair width; $L = 1/\sqrt{\rho}$ describes the average length of dislocation lines; ν_D is the Debye frequency; and k is the Boltzmann constant. Thus, at a certain strain rate $\acute{\varepsilon}$, a dislocation segment of length L glides with velocity $\bar{v}$ and efficiency of this motion at finite temperature depends on the kink-pair formation enthalpy $\Delta H_{2k}(\sigma)$. In this study, evolution of the latter with applied stress is acquired from the LT model applied for [100](010) screw dislocations, relying on the results of the performed atomic-scale modeling. The $\Delta H_{LT}(\sigma)$ curves (see Fig. 3b) are further parameterized using the continuous formulation

$$\Delta H_{LT}(\sigma) = 2H_k\left(1-\left(\frac{\sigma}{\sigma_p}\right)^p\right)^q, \qquad (S2)$$

where σ_p is the Peierls stress; p and q are adjustable parameters; and $2H_k(\sigma{=}0)$ is the total activation enthalpy, corresponding to two times the energy of an isolated kink at no stress applied.

Relying on the equations (S1) and (S2), one can express the critical resolved shear stress (CRSS) as the function of temperature:

$$\sigma = \sigma_p\left(1-\left(C\frac{T}{2H_k}\right)^{1/q}\right)^{1/p} = \sigma_p\left(1-\left(\frac{T}{T_a}\right)^{1/q}\right)^{1/p}, \qquad (S3)$$

where $C = ln\frac{2\acute{\varepsilon}w^2}{\sqrt{\rho}a' b^2\nu_D}$ is a function of a strain rate $\acute{\varepsilon}$, dislocation density ρ, and the kink geometry: width w and height a'; T_a is the so-called athermal temperature at which lattice friction of a material vanishes. From the performed DFT simulations, $2H_k$ = 2.69 eV, p = 0.73 and q = 1.31 are defined. Considering experimental conditions, *i.e.* strain rates $\acute{\varepsilon}$ = ~10^{-5} s^{-1} and dislocation density ρ = 10^{12} m^{-2}, we find that for [100](010) system in $MgSiO_3$ post-perovskite lattice friction vanishes if the temperature is raised above 1,100 K (see Fig. S5).

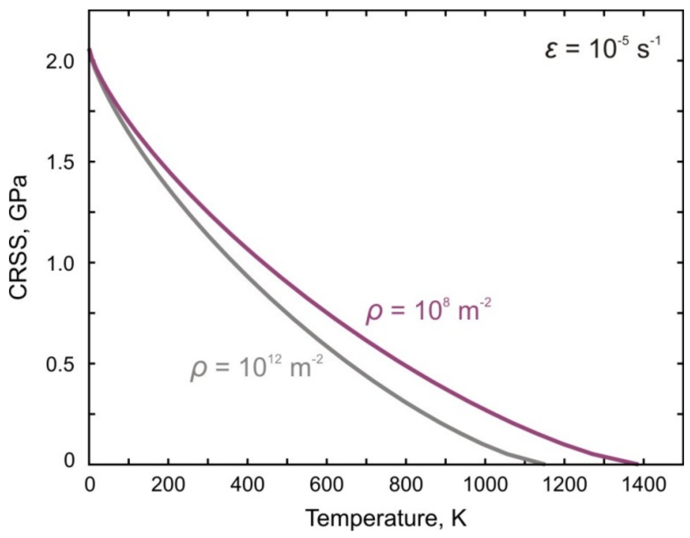

Figure S5. Evolution of critical resolved shear stress (CRSS) with temperature computed for [100](010) system in $MgSiO_3$ post-perovskite at 120 GPa and experimental strain rate $\dot{\varepsilon} = 10^{-5}$ s^{-1} based on the data from DFT simulations. Dislocation density ρ is provided on the plot for each curve

References

Bigger, J.R.K., McInnes, D.A., Sutton, A.P., Payne, M.C., Stich, I., King-Smith, R.D., Bird, D.M. & Clarke, L.J. Atomic and electronic structures of the 90° partial dislocation in silicon. *Phys. Rev. Lett.* **69**, 2224–2227 (1992)

Clouet, E. Dislocation core field. I. Modeling in anisotropic linear elasticity theory. *Phys. Rev. B* **84,** (2011)

Goryaeva, A.M., Carrez, Ph. & Cordier, P. Modeling defects and plasticity in $MgSiO_3$ post-perovskite: Part 2 – screw and edge [100] dislocations. *Phys. Chem. Miner.* **42**, 793-803 (2015)

Henkelman, G. & Jónsson, H. Improved tangent estimate in the nudged elastic band method for finding minimum energy paths and saddle points, *J. Chem. Phys.* **113**, 9978-9985 (2000)

Henkelman, G., Uberuaga, B.P. & Jónsson, H. A climbing image nudged elastic band method for finding saddle points and minimum energy paths, *J. Chem. Phys.* **113**, 9901-9904 (2000)

Nakano, A. A space–time-ensemble parallel nudged elastic band algorithm for molecular kinetics simulation. *Comp. Phys. Comm.* **178**, 280-289 (2008)
